# How reliable is the linear noise approximation of gene regulatory networks?

**DOI:** 10.1186/1471-2164-14-S4-S5

**Published:** 2013-10-01

**Authors:** Philipp Thomas, Hannes Matuschek, Ramon Grima

**Affiliations:** 1 School of Biological Sciences, University of Edinburgh, UK; 2 School of Mathematics, University of Edinburgh, UK; 3 Institute of Physics and Astronomy, University of Potsdam, Germany

## Abstract

**Background:**

The linear noise approximation (LNA) is commonly used to predict how noise is regulated and exploited at the cellular level. These predictions are exact for reaction networks composed exclusively of first order reactions or for networks involving bimolecular reactions and large numbers of molecules. It is however well known that gene regulation involves bimolecular interactions with molecule numbers as small as a single copy of a particular gene. It is therefore questionable how reliable are the LNA predictions for these systems.

**Results:**

We implement in the software package intrinsic Noise Analyzer (iNA), a system size expansion based method which calculates the mean concentrations and the variances of the fluctuations to an order of accuracy higher than the LNA. We then use iNA to explore the parametric dependence of the Fano factors and of the coefficients of variation of the mRNA and protein fluctuations in models of genetic networks involving nonlinear protein degradation, post-transcriptional, post-translational and negative feedback regulation. We find that the LNA can significantly underestimate the amplitude and period of noise-induced oscillations in genetic oscillators. We also identify cases where the LNA predicts that noise levels can be optimized by tuning a bimolecular rate constant whereas our method shows that no such regulation is possible. All our results are confirmed by stochastic simulations.

**Conclusion:**

The software iNA allows the investigation of parameter regimes where the LNA fares well and where it does not. We have shown that the parametric dependence of the coefficients of variation and Fano factors for common gene regulatory networks is better described by including terms of higher order than LNA in the system size expansion. This analysis is considerably faster than stochastic simulations due to the extensive ensemble averaging needed to obtain statistically meaningful results. Hence iNA is well suited for performing computationally efficient and quantitative studies of intrinsic noise in gene regulatory networks.

## Background

It is generally accepted that the relative size of molecular fluctuations scales as the inverse square root of the mean molecule numbers [1]. Since the key players of gene regulatory networks are present in amounts as small as one molecule it follows that gene expression is inherently noisy [2,3]. This molecular noise manifests itself in the copy number variations of transcripts and their proteins among genetically identical cells [4]. The main measures that have been used to quantify these cell-to-cell variations both experimentally and through modeling are the coefficient of variation (CV) and the Fano factor [5-9].

Exact analytical results for these quantities have been derived only for very simple gene regulatory systems [10-12] and hence they are more commonly obtained by means of Monte Carlo simulations using the stochastic simulation algorithm (SSA) [13,14]. Despite being formally exact with the Chemical Master Equation (CME), in practice, this approach turns out to be computationally expensive mainly due to the considerable amount of sampling required to compute reliable statistical averages. The situation is exacerbated when networks are to be studied over a wide range of parameters. The main analytical tool to address this issue has since been the linear noise approximation (LNA) of the Chemical Master Equation (CME) [15-17] which allows one to approximate the dynamics of the latter by a set of linear stochastic differential equations from which all moments can be computed in closed form. In this approximation, the mean concentrations of the CME are approximated by the solution of the deterministic rate equations (REs) and the probability distribution of the fluctuations is approximated by a Gaussian. Thereby the LNA can give insight into the parametric dependence of the noise whenever the REs admit a unique steady state solution. However unlike the CME, this approximation is valid only in the limit of large molecule numbers and hence the accuracy of its predictions is questionable for intracellular biochemical reaction networks [18,19]. A handful of theoretical studies access the accuracy of the REs and the LNA predictions by computing finite molecule number corrections to both approximations [20-22], a task which can be carried out analytically only for some simple systems. Hence, to-date, it is unclear how important these corrections are for many gene regulatory networks of interest.

We recently developed intrinsic Noise Analyzer (iNA) [23], the first software package enabling a fluctuation analysis for a broad class of biochemical networks of interest via the LNA and the Effective Mesoscopic Rate Equation (EMRE) approximations of the CME. The latter approximation gives accurate mean concentrations for systems characterized by intermediate to large molecule numbers and is hence more accurate than the conventional REs.

In this article we develop and efficiently implement in iNA, the Inverse Omega Square (IOS) method which gives the variances and covariances of fluctuations about the means calculated by the EMRE method. From these we can calculate the CVs and Fano factors of mRNA and protein fluctuations to an accuracy higher than possible with the LNA. Hence the software iNA provides a means of probing the validity of the LNA for any biochemical network under study. We use the EMRE and IOS methods to study the parametric dependence of the CV and Fano factors of mRNA and protein fluctuations in two examples of stochastic gene regulation involving nonlinear protein degradation, post-transcriptional, post-translational and negative feedback regulation. We show that these results agree with stochastic simulations but in many instances disagree with the LNA results. In particular the LNA predicts that the noise levels can be optimized by tuning a bimolecular rate constant whereas no such regulation is predicted by EMRE/IOS and simulations. It is also found that the LNA significantly underestimates the amplitude and period of noise-induced oscillations in genetic oscillators. Using detailed benchmarks we demonstrate that the present methodology is typically computationally more efficient than stochastic simulations using the SSA.

## Results

In this section we describe the results of the novel IOS method implemented in iNA. Its predictions are compared to the RE and LNA approximations of the CME and with exact stochastic simulations using the SSA for two examples of gene regulation. Finally we discuss its computational efficiency. The three methods (LNA, EMRE, IOS) are obtained from the system size expansion of the CME [15] which is applicable to monostable chemical systems. Technical details of the various approximation methods are provided in the section Methods.

### Investigating the parametric dependence of the size of molecular fluctuations

Biochemical reactions occur in random order and at random time. The stochastic description of biochemical reaction kinetics considers *N *distinct chemical species confined in a volume Ω reacting via *R *chemical reactions of the form

(1) $$s_{1j}X_{1}+\ldots +s_{Nj}X_{N}\overset{k_{j}}{\to }r_{1j}X_{1}+\ldots +r_{Nj}X_{N},$$

where *j *varies from 1 to *R*. The mesoscopic state of the system is given by the vector of molecular populations $\vec{n}=\left(n_{1},n_{2},\ldots ,n_{N}\right)$ and can be characterized by the probability $P\left(\vec{n}\right)$ to find the system in a particular configuration $\vec{n}$. The latter is however very difficult to obtain from analysis and hence intrinsic noise may be more easily characterized in terms of CVs and Fano factors which are defined in the following. We show how these quantifies are calculated using the LNA and higher order approximations implemented in the software iNA.

The CV of the number of molecules of species *X_i _*is defined by

(2) $$C_{V,i}=\frac{\sqrt{\text{Var}\left(n_{i}\right)}}{\text{E}\left(n_{i}\right)},$$

where Var(*n_i_*) denotes the variance and E(*n_i_*) the expectation value of the number of molecules of species *X_i_*. The CV quantifies the relative spread about the mean or put in different terms, it measures the inverse signal-to-noise ratio. Because of the latter fact it is often referred to as the "size" of the noise. A different but commonly used noise measure is the Fano factor defined by

(3) $$F_{i}=\frac{\text{Var}\left(n_{i}\right)}{\text{E}\left(n_{i}\right)}.$$

The Fano factor allows one to compare the spread of probability distributions relative to a Poissonian with the same mean. Notice that both quantifiers are determined solely by knowledge of the means and variances which can be obtained as a power series in the inverse compartment volume Ω by van Kampen's system size expansion of the master equation [15]. This expansion is carried out at constant concentration and hence the large volume limit is equivalent to the limit of large molecule numbers. As shown in the section Methods, the expansion's leading order term (Ω^0^) for the mean concentrations is given by the REs and the next to leading order term (Ω^-1^) by the EMREs. Similarly the variances about these concentrations are given to leading order (Ω^-1^) by the LNA and the next to leading order term (Ω^-2^) by what we here refer to as the IOS approximation. It then follows that the CV has the expansion

(4) $$C_{V,i}=C_{V,i}^{\text{LNA}}\left(\vec{k},\Omega \right)\left(1+\Omega^{-1}c_{i}\left(\vec{k}\right)+O\left(\Omega^{-2}\right)\right),$$

and a similar expansion holds for the Fano factor

(5) $$F_{i}=F_{i}^{\text{LNA}}\left(\vec{k}\right)\left(1+\Omega^{-1}f_{i}\left(\vec{k}\right)+O\left(\Omega^{-2}\right)\right).$$

The above expressions fully characterize the parametric dependence of the size of the fluctuations in terms of the compartment volume Ω, the set of reaction rate constants $\vec{k}=\left(k_{1},k_{2},\ldots ,k_{R}\right)$ and the set of initial conditions whose explicit dependence has been omitted here. Note that the leading order contributions in the infinite volume limit, $C_{V,i}^{\text{LNA}}\left(\Omega ,\vec{k}\right)$ and $F_{i}^{\text{LNA}}\left(\vec{k}\right)$, are given by the LNA's result for the CV and Fano factor which can be shown to scale as Ω^-1/2 ^and Ω^0^, respectively. The factors $c_{i}\left(\vec{k}\right)$ and $f_{i}\left(\vec{k}\right)$ determine the relative corrections to the LNA result and can be obtained from the EMRE and IOS approximations as has been carried out explicitly in the Methods section. It can be argued that the size of these correction terms is proportional to the bimolecular reaction rate constants since the LNA is exact up to second moments for networks composed solely of unimolecular reactions since the propensities are linear functions of the concentrations (see section Methods). Summarizing, this analysis suggests novel correction terms to the CVs and Fano factors that are of order Ω^-3/2 ^and Ω^-1^, respectively, and hence of higher accuracy than the LNA.

**Figure 1:**
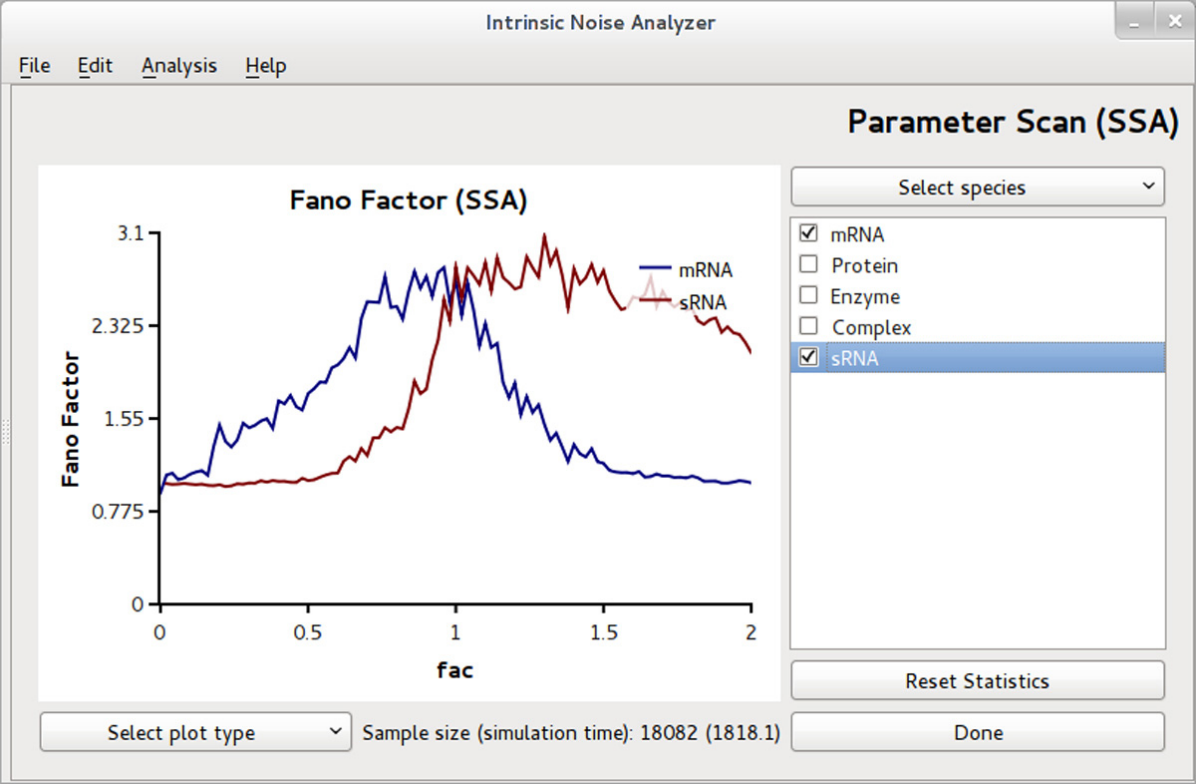
**SSA parameter scan dialog with online statistics**. iNA allows the acquisition of time-averaged statistics from the SSA within a user-friendly dialog. This type of analysis is applicable under stationary conditions and provides mean, variance, CVs and Fano factor statistics over a wide range of parameters.

Complementary to the LNA and IOS analysis the noise coefficients can be obtained by stochastic simulations using the SSA. Although this method is formally exact we note that noise estimators may be strongly biased for small to intermediate sample sizes [24]. The large amount of ensemble averaging required makes it computationally expensive to obtain these estimates from stochastic simulations. A commonly used method to accelerate the statistical averaging procedure involves replacing the ensemble average by a time average which is allowed under steady state conditions and the assumption of ergodicity of sample paths [25]. In particular, using the SSA one time-averages over a sufficiently long time series to estimate the noise coefficients. The present version of iNA facilitates this procedure by computing the stationary moments via a time-averaged SSA. A convenient on-the-fly dialog allows one to remove transients from simulated trajectories and to monitor the convergence of mean, variance, CV or Fano factor statistics. In Figure 1 we present a screenshot of this dialog.

### Applications

While gene expression is a complex process the most commonly used model is naturally the simplest. The model describes the transcription of mRNA and the translation of proteins from mRNA and the subsequent degradation by the effective first order reactions:

(6) $$\begin{array}{l} \text{Gene}\to \text{Gene}+\text{mRNA},\\ \text{mRNA}\to \text{mRNA}+\text{Protein},\\ \text{Protein}\to \emptyset ,\;\text{mRNA}\to \emptyset . \end{array}$$

The model has been used to quantify variability in the proteome of *E. coli *[3,5,10], yeast [26] and mammalian cells [27] as well as having being subject to a number of theoretical studies [6,11]. The LNA's predictions of the first two moments of this model are exact since it is composed of only unimolecular reactions.

Given the complexity of the intracellular biochemistry, it is clear that this simple model cannot fully account for regulation which occurs at transcriptional, post-transcriptional, translational and post-translational stages. These processes typically involve bimolecular reactions with regulatory molecules such as transcription factors, functional RNAs or enzymes. While it is obvious that the CVs and Fano factors of more realistic models will differ from those predicted by the "standard" linear model (6), it is however not immediately clear whether these noise measures are qualitatively different than those obtained from the LNA.

In this section we demonstrate the use of the software iNA, which makes use of the approximation methods described in the previous section, to predict the noise characteristics of two gene regulatory networks involving post-transcriptional regulation by non-coding RNA and negative autoregulation via post-translational modification. Specifically we focus on how well these characteristics are described by the LNA both quantitatively and qualitatively and point out the LNA's limitations using correction terms of the IOS analysis and stochastic simulations provided by iNA.

#### sRNA mediated post-transcriptional regulation

A large number of functional RNAs called small RNAs (sRNAs) have been found in bacteria which are not actively translated. This non-coding form of RNA is believed to coordinate pathways in response to external stimuli such as stress [28,29]. To investigate the robustness of critical pathways it is therefore important to understand the impact of intrinsic noise on their regulation.

Here we extend the mechanism of gene expression with nonlinear degradation studied in [30] to include the regulation of gene expression by sRNA in response to stress. A generic model of non-catalytic sRNA-mRNA interaction is

(7a) $$\begin{array}{l} \text{Gene}\overset{k_{0}}{\underset{}{\to }}\text{Gene}+\text{mRNA},\;\text{mRNA}\overset{k_{\text{dM}}}{\underset{}{\to }}\emptyset ,\\ \text{mRNA}\overset{k_{s}}{\underset{}{\to }}\text{mRNA}+\text{Protein,}\\ \text{Protein}+\text{Enzyme}\overset{k_{1}}{\underset{k_{-1}}{⇌}}\text{Complex}\overset{k_{2}}{\underset{}{\to }}\text{Enzyme}+\emptyset , \end{array}$$

(7b) $$\begin{array}{l} \text{Gen}{\text{e}}^{*}\overset{k_{0}\alpha }{\underset{}{\to }}\text{Gen}{\text{e}}^{*}+\text{sRNA},\;\text{sRNA}\overset{k_{\text{ dS}}}{\underset{}{\to }}\emptyset ,\\ \text{sRNA}+\text{mRNA}\overset{k_{R}}{\underset{}{\to }}\emptyset . \end{array}$$

Note that reactions (7a) are as considered in Ref. [30]. In (7b) we describe the transcription and degradation of sRNA with respective rates *k*_0*α *_and *k*_dS_. The parameter *α *is given by the ratio of sRNA to mRNA transcription and can be used to describe the coordination of the stress response due to tight regulation of sRNA transcription. When sRNA is expressed it binds with its mRNA target at a rate *k_R _*and quickly degrades thereafter. Similar models have been studied in Refs. [31,32].

**Figure 2:**
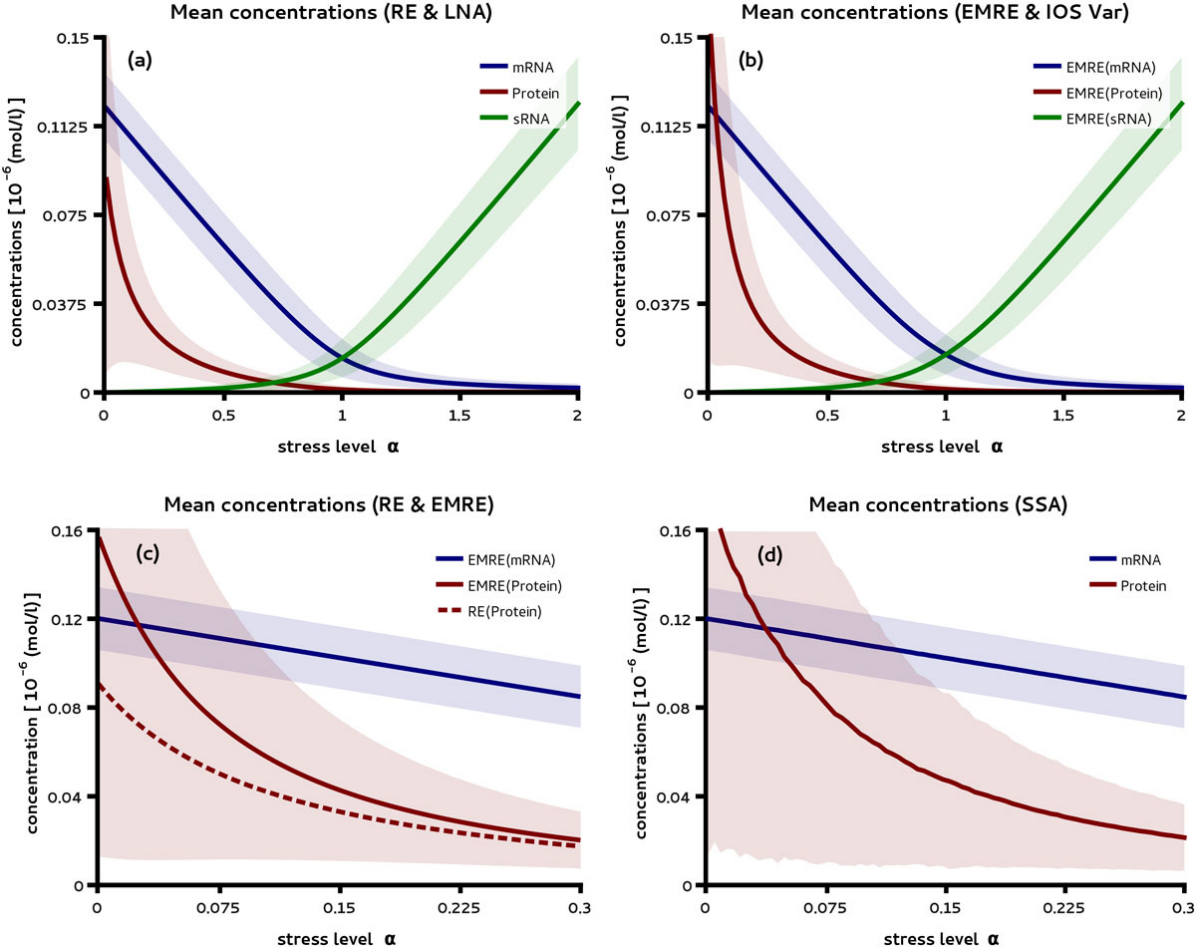
**Threshold responses of mRNA and protein concentrations under sRNA regulation**. We compare the mean concentrations predicted by the LNA method as a function of the parameter *α *shown in (a) with those predicted by the more accurate EMREs shown in (b). The average expression levels predicted by both methods are in good agreement except for small *α *(c) which is verified by stochastic simulations using the SSA (d).

Understanding how pathways are regulated in the presence of noise requires to study their response over a wide range of parameter values. Such a task is typically computationally expensive when carried out by stochastic simulations. We used iNA to investigate the impact of stress on our gene regulatory network both using the system size expansion and the SSA method. To our knowledge the effect of sRNA regulation on protein noise with a nonlinear degradation mechanism has not been studied before. Using parameter set (i) in Table 1 with iNA, we obtained the mean concentrations and standard deviations estimated from the REs and the LNA, respectively; these are shown in Figure 2 (a). Similarly the EMRE concentrations and IOS standard deviations for the same parameters are shown in (b). As stress levels are increased the characteristic threshold response is observed, i.e., as the expression level of sRNA rises it down-regulates the target mRNA concentrations [29]. Note that for *α <*1 the protein is expressed while it is turned off for *α >*1 while at the crossover point (*α *= 1) the levels of sRNA and mRNA are equal. Depending on the relative abundance of sRNAs and mRNAs in unstressed cells protein expression may be activated or silenced. A comparison of Figure 2 (a) and (b) shows that the predictions of the RE/LNA and of the EMRE/IOS agree well over large ranges of *α*. The two differ for small *α*, i.e., for small stress, (see Figure 2 (c)) where REs predict the mRNA levels to be higher than the protein ones while the EMRE method predicts the opposite. This phenomenon has been called discreteness-induced inversion [19] and has been discussed for the unregulated case in [30]. The effect is validated by stochastic simulations in Figure 2 (d). It is interesting that this inversion disappears for minute concentrations of sRNA corresponding to less than a single transcript and hence shows that the REs are surprisingly accurate over a wide range of stress levels.

**Table 1:** Gene expression model with sRNA regulation

paramater	set(i)	set (ii)
*k*_0_[*G*]	0.024min^-1^*µM*	0.0024min^-1^*µM*
*k*_dM_	0.2min^-1^	0.2min^-1^
*k_s_*	1.5min^-1^	1.5min^-1^
*k*_-1_, *k*_2_	2min^-1^	2min^-1^
*k*_1_	400(*µM *min)^-1^	4000(*µM *min)^-1^
*k*_dS_	0.2min^-1^	0.2min^-1^
*k_R_*	100(*µM *min)^-1^	1000(*µM *min)^-1^

Kinetic parameters used for the gene expression model with sRNA regulation, scheme (7), as discussed in the main text. The volume is fixed to 10^-15^*l *with total enzyme concentration of 0.1*µM *for parameter set (i) and 0.01*µM *for parameter set (ii) corresponding to 60 and 6 enzyme molecules, respectively. Note that [*G*] denotes the constant gene concentration.

**Figure 3:**
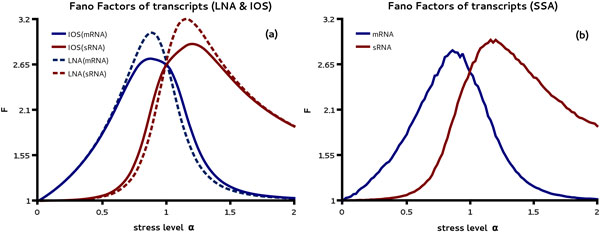
**Stress level dependence of Fano factors for coding and non-coding transcripts**. In (a) we compare the Fano factors (F) of transcripts predicted by the LNA as a function of the parameter *α *with those predicted by the IOS method. Comparing (a) with the results of the SSA shown in (b) we see that both methods, LNA and IOS, are in qualitative agreement with the stochastic simulations. However, the IOS method matches more closely the maximum Fano factors obtained from stochastic simulations.

Next we study the dependence of the CVs and Fano factors of coding and non-coding transcripts on the stress level. The mean and variances shown in Figure 2 can be used to compute both of these quantities. Here the Fano factor is of particular interest since in the absence of sRNA control, the molecule number of mRNA is exactly Poissonian distributed with Fano factor one [11]. Hence the Fano factor can be used to study the impact of regulation on the noise. In Figure 3 (a) it is shown that the Fano factor of transcripts is increased shortly before and after the crossover point for mRNA and sRNA, respectively. This highlights an increase of the mRNA noise levels by sRNA regulation in comparison to the situation where the same average mRNA concentration is obtained by regulating its transcription rate. Interestingly we find that the LNA prediction for the Fano factors of transcripts near their peak values are larger than those from the IOS. This over-estimation by the LNA is confirmed by stochastic simulations shown in Figure 3 (b). Note that while the Fano factors of the transcripts have a peak for intermediate stress levels the dependence of the associated CVs is monotonous (Additional file 1).

**Figure 4:**
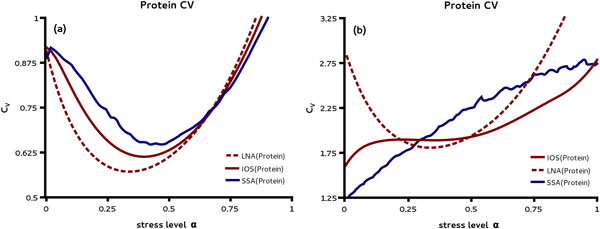
**Protein noise is minimized at intermediate stress levels**. Protein CV is shown for moderate (a) and low protein copy numbers (b) as a function of the stress level *α*. In (a) we compare the LNA estimates with those of the IOS method for moderate copy numbers using parameter set (i) in Table 1. Both methods predict that the CV reaches a minimum below the crossover point where the protein expression is activated. The prediction is qualitatively confirmed by the SSA. Notice that the IOS method predicts larger values for the minimum CV and larger values of *α *at which it is attained; this is also observed using the SSA. In (b) we compare the CV of protein concentration obtained using the LNA and IOS methods for low copy numbers using parameter set (ii) in Table 1. The LNA shows the same dependence as in (a) with increased noise levels. In contrast, the IOS method predicts a monotonic increase in the noise levels as the stress level is increased. The predictions of the IOS method are qualitatively confirmed by stochastic simulations.

In order to investigate the impact of stress on protein noise levels we analyzed the CVs of protein noise as predicted by the LNA and the IOS theory. The result is shown in Figure 4 (a). We find a minimum of the noise coefficient for intermediate levels of stress in the activated regime. Comparing the result to stochastic simulation also shown in Figure 4 (a) we see that both approximations yield the same qualitative result but the IOS coefficient is slightly more accurate in predicting the position and value of the minimum. Note that here the protein level corresponds to a copy number of about 60 molecules.

Genome-wide studies in *E. coli *revealed that some proteins can be expressed in much lower copy numbers than 60 [3]. We next make use of parameter set (ii) in Table 1 to probe the validity of the LNA under low copy number conditions. The results for the CV are shown in Figure 4 (b). In the absence of sRNA control the protein levels correspond to approximately 6 protein molecules. We observe that for such low copy numbers the LNA is in severe qualitative disagreement with the IOS. In particular, the LNA predicts that there exists a stress level for which the size of the noise is minimized while, in contrast, the IOS predicts the noise level to increase monotonically with stress. The latter is also reproduced by simulations using the SSA in Figure 4 (b) which hence signals the breakdown of the LNA under low copy number conditions.

#### Gene expression with negative autoregulation

Autoregulation represents a common mechanism by which cells regulate their expression levels. Specifically in *E. coli *about 35% - 43% of transcription factors are autoregulated [33,34] while in *S. cerevisiae *these account for about 10% [35]. We consider the common model of transcription and translation

(8) $$\begin{array}{c} G\overset{k_{0}}{\underset{}{\to }}G+M,\;M\overset{k_{\text{dm}}}{\underset{}{\to }}\emptyset ,\\ M\overset{k_{s}}{\underset{}{\to }}M+P,\;P\overset{k_{\text{dp}}}{\underset{}{\to }}\emptyset , \end{array}$$

where *G*, *M *and *P *refer to gene, mRNA and protein species, respectively. The first order degradation reactions here may also account for dilution due to cell growth. Next we add a negative feedback loop via a reversible phosphorylation mechanism and consequent transcriptional repression. Protein phosphorylation is common to post-translational regulation mechanisms [36-39] as for instance the negative feedback loop of the Drosophila circadian rhythm [40]. In fact, the majority of phosphorylated proteins in yeast are transcription factors [41]. The modification is modeled explicitly via a protein kinase *K *and a phosphatase *R *as follows

(9) $$\begin{array}{c} K+P\overset{k_{1}}{\underset{k_{-1}}{⇌}}KP\overset{{k^{\prime }}_{1}}{\underset{}{\to }}K+P_{*},\\ R+P_{*}\overset{k_{2}}{\underset{k_{-2}}{⇌}}RP_{*}\overset{{k^{\prime }}_{2}}{\underset{}{\to }}R+P, \end{array}$$

where P_* _denotes the phosphorylated form of protein which can bind to the DNA and thereby inhibits its own expression.

(10) $$\begin{array}{c} P_{*}+G\overset{k_{3}}{\underset{k_{-3}}{⇌}}GP_{*},\\ P_{*}+GP_{*}\overset{k_{4}}{\underset{k_{-4}}{⇌}}GP_{*}^{2}. \end{array}$$

Note that unlike the previous case of post-transcriptional regulation, here the promoter can be in one of three states *G*, GP_* _and $GP_{*}^{2}$ depending on the number of bound protein molecules. The degradation as in the preceding example is assumed to occur via two proteases *E *and *D*

(11) $$\begin{array}{c} P+E\overset{k_{5}}{\underset{k_{-5}}{⇌}}EP\overset{{k^{\prime }}_{5}}{\underset{}{\to }}E,\\ P_{*}+D\overset{k_{6}}{\underset{k_{-6}}{⇌}}DP_{*}\overset{{k^{\prime }}_{6}}{\underset{}{\to }}D. \end{array}$$

A similar model has been analyzed using the LNA and the EMREs implemented in a previous version of iNA in Ref. [23]. With the present version of iNA the more accurate IOS analysis is available and is used here to investigate the reliability of the LNA estimates of the CVs. This presents a major benchmark for the LNA since the analysis includes fluctuations of a single promoter.

**Figure 5:**
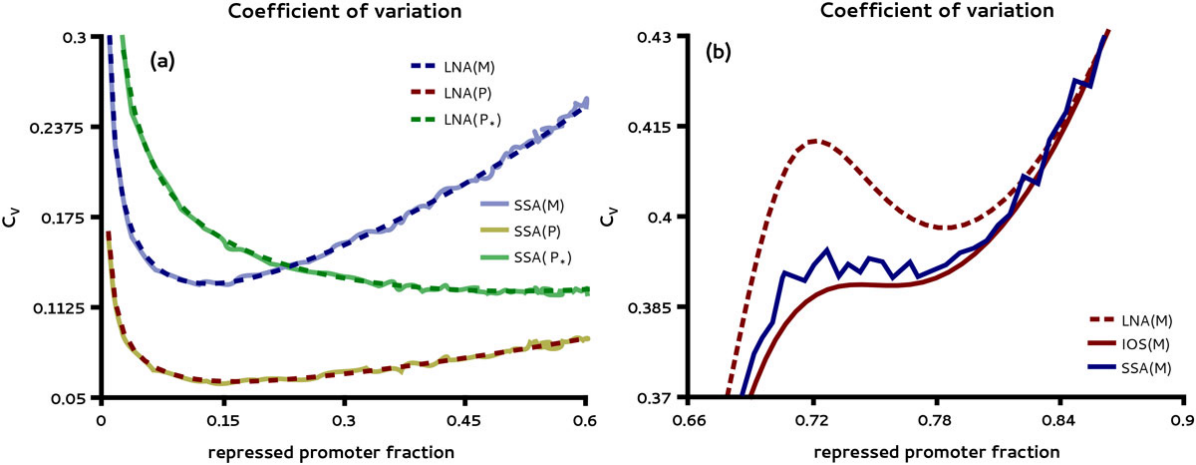
**CV of mRNA and protein fluctuations in gene autoregulation**. In (a) CVs of mRNA and protein levels are shown as function of the average repressed promoter fraction by variation of the transcription rate *k*_0_. The result obtained using the LNA indicates that the CVs of mRNA and unphosphorylated protein are minimized when the repressed promoter fraction is small (10 - 15%) which is in good agreement with stochastic simulations. In (b) we compare the mRNA CV obtained using the LNA with the IOS as function of the average occupation of repressed promoters varied through the protein-DNA association rate *k*_3 _which is a measure of feedback strength. While the LNA predicts the CV to have a maximum for moderately repressed promoters the IOS analysis shows a monotonically increasing dependence on the feedback strength with significantly smaller noise levels. The latter is confirmed by stochastic simulations.

We start by exploring the dependence of mRNA and protein CVs by varying the transcription rate *k*_0 _of the promoter. Using the LNA for the parameter set given in Table 2 we calculated the CV as a function of the average fraction of repressed promoter states. The latter is given by the sum of the average occupation of the protein bound promoter states GP_* _and $GP_{*}^{2}$ and hence represents a measure of the feedback strength. We observe that both mRNA and the unphosphorylated protein CVs are minimized for small values of the feedback strength as shown in Figure 5 (a). These predictions are in excellent agreement with the results of the SSA. Next we investigate the parametric dependence of the fluctuations for larger values of the feedback strength varied through the rate constant *k*_3 _of the protein-DNA association rather than the transcription rate. In Figure 5 (b) we show that the LNA predicts that the mRNA CV has a non-monotonic dependence on the average fraction of repressed promoter states. In contrast to the LNA, the IOS analysis predicts the CV to be a monotonically increasing function of the feedback strength showing no maximum. We have verified this dependence by stochastic simulations also shown in Figure 5 (b). In contrast to the mRNA CV, the same analysis carried out for the CVs of proteins yields qualitative agreement between LNA and SSA (Additional file 2).

**Table 2:** Genetic expression model with negative autoregulation

parameter	Value	parameter	value
*k*_0_	50$\tilde{\Omega }$ (*nM*)	*k_s_*	50*h*^-1^
*k*_dM_	5*h*^-1^	*k*_dp_	0.5*h*^-1^
*k*_1_	0.5 · (*nMh*)^-1^	*k*_-1_	1*h*^-1^
*k*_2_	0.5 · (*nMh*)^-1^	*k*_-2_	*k*_-1_
*k*_3_	0.5$\tilde{\Omega }$	*k*_-3_	450*h*^-1^
*k*_4_	50 · *k*3	*k*_-4_	*k*_-3_
*k_5_*	0.25 · (*nMh*)^-1^	*k*_-5_	0.5*h*^-1^
*k_6_*	5 · (*nMh*)^-1^	*k*_-6_	5*h*^-1^
$k_{6}^{\prime }$	10*h*^-1^	$k_{5}^{\prime }$	0.5*h*^-1^
$\tilde{\Omega }$	450 · (*nM*)^-1^		

Kinetic parameters for the model of autoregulated gene model for a volume of 7.5 × 10^-13^*l*. Note that here the parameter $\tilde{\Omega }$ is the inverse concentration of a single molecule. The total enzyme concentration of *E*, *K*, *R *is given by 0.5*nM *while the concentration of *D *is 5*nM*.

This result suggests that the corrections to the LNA are susceptible to the fluctuations in the promoter states. In order to test this hypothesis we investigated the oscillatory dynamics (that is often associated with the presence of a negative feedback loop) as a function of the gene copy number. In rapidly growing *E. coli*, for instance, the copy number of chromosomal genes located near the origin of DNA replication can be increased by 4-fold over genes located near the terminus [42]. Moreover genes located on plasmids can be present in higher copy numbers than those integrated in the genome. For synthetic circuits the plasmid copy number can also be controlled experimentally [43,44].

**Figure 6:**
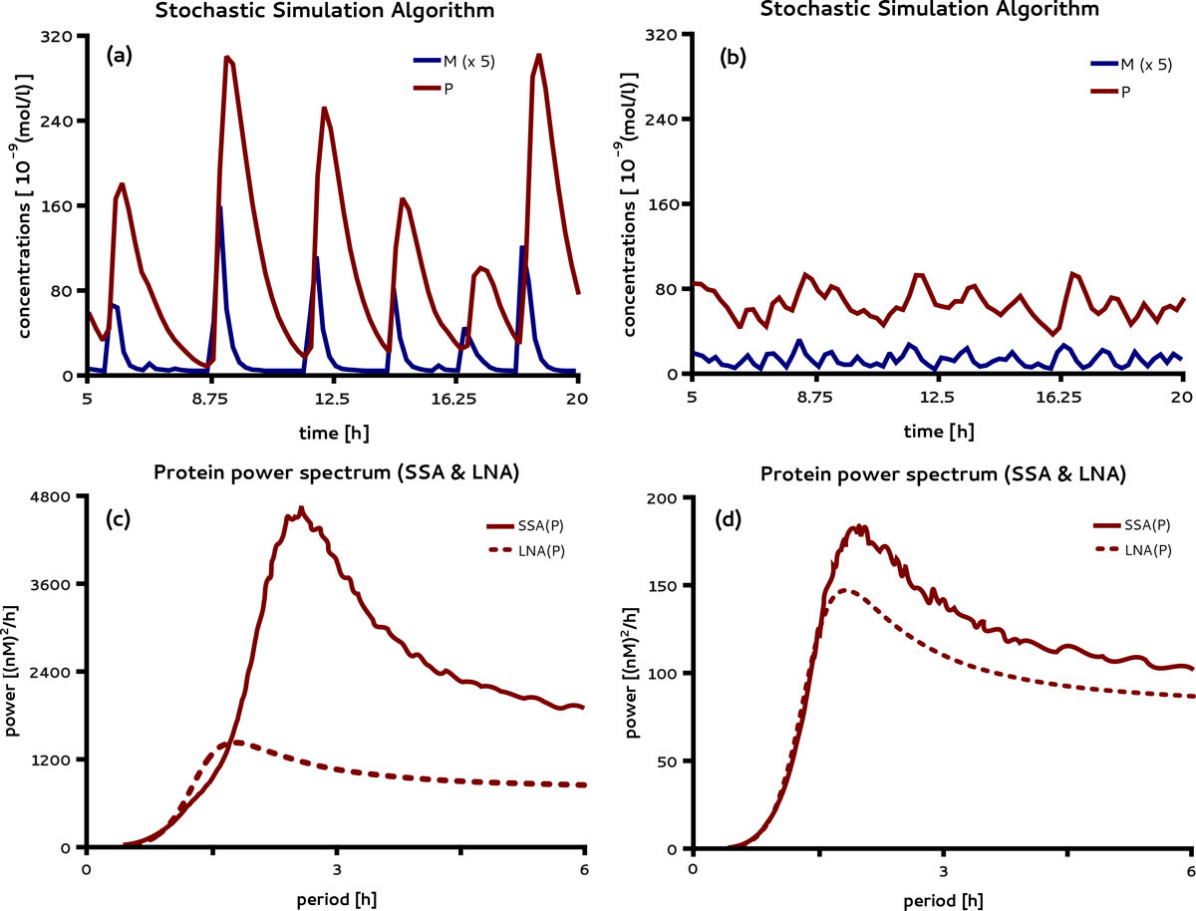
**Amplification of noise-induced oscillation by gene copy number control**. Highly periodic expression of mRNAs and proteins from a single promoter with negative feedback is shown in (a). In contrast the expression of 10 genes (b) using the same parameters attenuates the oscillations without apparent periodicities. The average power spectrum of the protein oscillations as obtained from the SSA and the LNA is shown for a single (c) and ten promoters (d). Note that while in (c) the LNA underestimates the amplitude of the oscillations it also fails to accurately predict their period. The power spectrum in (d) reveals the weak oscillatory behavior of the time traces in (b) in good agreement with the SSA. The parameters used are given in Table 2 except for a volume of 50 × 10^-15^*l *with $\tilde{\Omega }=\text{ 3}0{\left(nM\right)}^{-\text{1}}$ and $k_{0}=3\times 10^{3}N_{G}^{-1}{\left(nM\right)}^{-1}$ where *N_G _*is the gene copy number.

Variation of the copy number typically yields elevated protein concentrations if dosages are not compensated. For simplicity, here we scaled the transcription rate *k*_0 _by the number of genes which yields the same steady state expression levels for the deterministic REs independent of the gene copy number. Figure 6 (a) shows the oscillatory protein expression of a single gene obtained from a SSA realization in a reduced volume of 5 × 10^-14^*l *which roughly corresponds to that of yeast [45]. In Figure 6 (b) the same is shown for the expression of 10 genes. Notice that in contrast to the case of a single gene, the trajectories lack apparent periodicities. Hence we conclude that these oscillations are induced by limited gene copies. The signature of a noise-induced oscillation is a peak in the power spectrum of a system for which the deterministic REs show no sustained oscillations. In Additional file 3 we have verified that this is indeed the case. We next obtained the average power spectrum of the noise-induced oscillations from a large number of SSA realizations and compared it to the power spectrum that can be calculated from the LNA [46]. Figure 6 (c) and (d) show the power spectra of protein expression of a single gene and of 10 genes, respectively. We note that in both cases the LNA qualitatively captures the presence of noise-induced oscillations since the LNA spectra exhibit a peak at a non-zero frequency. However for the case shown in Figure 6 (c) both the oscillation amplitude (the square root of the peak power) and period are underestimated by about 50% percent using the LNA. In actuality, the dampening of single cell oscillations has been observed in synthetic circuits with varying plasmid copy number with similar shifts in their periods [47].

## Implementation

iNA is a GUI-based software which at heart is based on the SBML description of stoichiometric reaction networks. With the current release we introduce model manipulation capabilities which are tailored to fit the needs of stochastic modeling, as well as a just-in-time compilation engine that increases the overall execution speed of the analysis.

### Model editor capabilities

The software is based on the wide-spread SBML file format [48]. Although in common use, SBML has the shortcoming that it is barely human readable. The present version of iNA supports the compatible format SBML shorthand (SBML-sh) which represents the essential SBML model structure in an easy to read and write description language [49]. Therefore SBML-sh complements the existing SBML functionality by allowing import and export of both formats together with an online SBML-sh editor, see Additional file 4 (c).

Apart from this iNA's GUI also incorporates basic model editing capabilities. Additional file 4 (a) shows the list of reactions which allows to add or edit reactions within a dialog shown in Additional file 4 (b). Within this dialog the propensity of the reaction is either constructed automatically from the statistical formulation of the law of mass action [23,50,51] or to be specified by the user.

### Performance

The system size expansion of the CME yields a high dimensional system of coupled ODEs of order *N*^2 ^equations for the LNA and *N*^3 ^equations for the IOS analysis. Parameter scans as well as numerical integration of large systems are particularly challenging because of the large number of function evaluations needed to obtain accurate results. iNA's initial release addressed this issue by providing a bytecode interpreter for efficient expression evaluation [23]. The present version improves on this using a just-in-time (JIT) compiler provided by the LLVM infrastructure [30,52]. This technique provides the means of platform specific code generation for the system size expansion ODEs at runtime mimicking the performance of statically compiled code.

In order to access the performance of the present implementation we consider the model of negative autoregulation studied in the section Applications which involves 14 species and 20 reactions. After conservation analysis the model reduces to only 9 species which yields a total number of 273 simultaneous equations for the IOS method and it is hence well suited for direct benchmarking purposes. This is particularly challenging in terms of ODE integration since the mean couples to higher statistical moments and causes the full system of 273 coupled equations to exhibit damped oscillations (see Additional file 3). The results of the benchmarks are summarized in Table 3 highlighting the performance of the present version of iNA. The improvements of iNA's system size expansion using the LSODA algorithm [53] over the previous Rosenbrock method reduce the execution time by up to a factor of 10 using the JIT compiler. In comparison the overall execution of the SSA requires about half an hour and hence is computationally extremely expensive because of the considerable number of trajectories to be averaged in order to obtain accurate statistics.

**Table 3:** Performance of iNA's time course analysis

method	IOS, LSODA	IOS, Rosenbrock	SSA, single (ens.)
BCI	18.2s (18.5s)	59.5s (59.8s)	0.04s (0.5h)
JIT	0.9s (3.6s)	13.0s (35.8s)	0.03s (0.4h)

Execution times of iNA's timecourse analysis for IOS and SSA for the gene oscillatory network reproducing Additional file 3 (a) and (b), respectively. The table compares the IOS method using the ODE integrator LSODA and Rosenbrock in combination with a bytecode interpreter or iNA's JIT feature. The value in the brackets denotes the overall time including bytecode assembly or JIT compilation. The SSA value denotes the average time for a single run while the values in the brackets denote the extrapolated value for an ensemble of 50, 000 independent realizations needed to generate Additional file 3 (b). Benchmarks were performed on Ubuntu (64bit), Dell OptiPlex 9010 SF, Core i7-3770 @ 3.4GHz using a single core.

The analysis using the system size expansion is particularly advantageous when it is performed under steady state conditions because it can be readily obtained for large sets of parameters as we have shown in the section Applications. In this case the problem reduces to finding the solution of the 9 nonlinear deterministic REs and solving the remaining 264 linear equations (obtained from the system size expansion) from which the noise statistics are obtained. In Table 4 we summarize the detailed computation times for the REs, LNA and IOS analysis that have been employed to calculate the protein CVs (see Additional file 2 and Figure 5 (b)) showing the protein CVs. All analyses were performed in less than a second albeit the LNA is typically much quicker than the more accurate IOS method. For comparison we also show the average execution time per sample of the SSA using a finite sampling rate. We remark that the performance of the system size expansion methods could be increased by about 30% for the IOS and 40% for the SSA using iNA's integrated JIT compilation feature. We emphasize that the computation of the IOS analysis requires the same time as computing only 40 SSA samples. In particular to reproduce Additional file 2 and Figure 5 (b) a thousand-fold of this sample size was needed. The advantage of the IOS method as a complementary method to traditional means of stochastic simulation is therefore readily obvious.

**Table 4:** Performance of iNA's steady state parameter scan

method	REs	LNA	IOS	SSA per sample (overall)
GiNaC	0.61s	1.48s	39.93s	--
BCI	0.19s	0.19s	0.39s	11ms (16h)
JIT	0.17s	0.17s	0.28s	7ms (10h)

Execution times of iNA's parameter scan for the gene autoregulation network reproducing Additional file 2 (a) and (b), respectively. The table compares the REs, LNA, IOS and SSA method using the bytecode interpreter or iNA's JIT feature. For comparison we also show the execution time using the computer algebra system GiNaC which is limited by its arbitrary precision arithmetic. The SSA value denotes the average time per sample per parameter while the values in the brackets denote the extrapolated value for a sample size of 50, 000 and 100 sets of parameter values as has been used in Additional file 2. Benchmarks were performed on Ubuntu (64bit), Dell OptiPlex 9010 SF, Core i7-3770 @ 3.4GHz using a single core.

## Methods

We consider a reaction network confined in a volume Ω composed of *N *distinct chemical species reacting via *R *chemical reactions of the form

(12) $$s_{1j}X_{1}+\ldots +s_{Nj}X_{N}\overset{k_{j}}{\underset{}{\to }}r_{1j}X_{1}+\ldots +r_{Nj}X_{N}.$$

Here *j *is the reaction index running from 1 to *R*, *X_i _*denotes chemical species *i*, *k_j _*is the reaction rate constant of the *j^th ^*reaction and *s_ij _*and *r_ij _*are the stoichiometric coefficients. Let *n_i _*be the number of molecules of the *i^th ^*species. Under well-mixed conditions, the time evolution of the mesoscopic state $\vec{n}={\left(n_{1},\ldots ,n_{N}\right)}^{T}$ can be obtained either by Monte Carlo simulations using the SSA [14] or directly by determining the probability $P\left(\vec{n},t\right)$ of finding the system in a particular mesoscopic state using the CME

(13) $$\frac{\partial P\left(\vec{n},t\right)}{\partial t}=\Omega \overset{R}{\underset{j=1}{\sum }}\left({\hat{f}}_{j}\left(\vec{n}-{\vec{\mu }}_{j},\;\Omega \right)P\left(\vec{n}-{\vec{\mu }}_{j},\;t\right)-{\hat{f}}_{j}\left(\vec{n},\;\Omega \right)P\left(\vec{n},t\right)\right),$$

where ${\hat{f}}_{j}\left(\vec{n},\Omega \right)$ is the probability per unit time and unit volume for the *j^th ^*reaction to occur [14] and ${\left({\vec{\mu }}_{j}\right)}_{i}=r_{ij}-s_{ij}$ is the stoichiometry of species *i *in the *j^th ^*reaction. Note that both approaches are equivalent [51].

### Rate equations and the Linear Noise Approximation

The CME determines the probability of observing any combination of molecule numbers at any point in time. Hence for closed systems the state space grows exponentially with the number of species while for open systems it is generally infinite. It is this complexity which prevents one to obtain exact analytical solutions of the CME except in particular cases [11,12]. The most common approximation method is the LNA which has been derived by van Kampen through the system size expansion of the Master equation. In brief, the method separates the instantaneous concentration vector into a macroscopic part, $\left[\vec{X}\right]$, and the fluctuations $\vec{\varepsilon }$ about it:

(14) $$\frac{\vec{n}}{\Omega }=\left[\vec{X}\right]+\Omega^{-1/2}\vec{\varepsilon }.$$

The macroscopic part is obtained as the solution of the conventional REs

(15) $$\frac{\partial }{\partial t}\left[\vec{X}\right]=\overset{R}{\underset{j}{\sum }}{\vec{\mu }}_{j}f_{j}\left(\left[\vec{X}\right]\right).$$

The implicit assumption made by ansatz (14) is that in the infinite volume limit the instantaneous concentrations equal the solution of the REs. It can then be shown that the macroscopic rate function of the *j^th ^*reaction is obtained from the relation $f_{j}\left(\left[\vec{X}\right]\right)=\underset{\Omega \to \infty }{\text{lim}}{\hat{f}}_{j}\left(\left[\vec{X}\right],\;\Omega \right)$[30]. Since the limit is taken at constant concentration, this implies the large molecule number limit as well.

The method now proceeds by using the ansatz (14) together with Eq. (13) in order to obtain an equation for $\vec{\varepsilon }$. The result is an expansion of the CME in powers of the inverse square root of the volume which can be truncated. The first term in the expansion (Ω^1/2^) yields the REs. The next term (Ω^0^) is given by a linear Fokker-Planck equation called the linear noise approximation of CME. This approximation is in wide-spread use mainly because of the simplicity of the result: the LNA solution is given by a Gaussian distribution describing the fluctuations around the macroscopic concentrations predicted by the REs. Specifically, to the order of approximation this implies that the macroscopic equations determine the average concentrations. The covariance of fluctuations *σ_ij _*= 〈ε*_i_ε_j_*〉 then satisfies the following matrix equation [16]

(16) $$\underline{\text{J}}\;\underline{\sigma }\;\;+\;\underline{\sigma }\;{\underline{\text{J}}}^{T}\;+\;\underline{\text{D}}\;=\;0,$$

where $\underline{\text{J}}=\sum_{j=1}^{R}{\vec{\mu }}_{j}\nabla_{\left[\vec{X}\right]}^{T}f_{j}\left(\left[\vec{X}\right]\right)$ is the Jacobian of the macroscopic equations (15) and $\underline{\text{D}}=\sum_{j=1}^{R}{\vec{\mu }}_{j}{\vec{\mu }}_{j}^{T}f_{j}\left(\left[\vec{X}\right]\right)$ is the diffusion matrix. Using Eq. (14) we can write expressions for the CVs and Fano factor of the *i^th ^*species using the definitions (2) and (3) in the main text

(17) $$C_{V,i}^{\text{LNA}}=\Omega^{-1/2}\frac{\sqrt{\sigma_{ii}}}{\left[X_{i}\right]},F_{i}^{\text{LNA}}=\frac{\sigma_{ii}}{\left[X_{i}\right]},$$

which are of order Ω^-1/2 ^and Ω^0^, respectively.

It is well known that the means and variances of concentrations predicted by the LNA are exact only for reaction networks involving at most unimolecular reactions. For bimolecular reactions the LNA can be inaccurate if some species are present only in low molecule numbers. This is because for unimolecular reactions the hierarchy of moment equations obtained from the CME is closed, i.e., the *n^th ^*moment depends only the (*n *- 1)*^th ^*moment and all lower order moments [54]. The equations for the first moment are given by the REs since the propensities are linear functions of the concentrations. The equations for the second moments are a system of linear equations for the variances and covariances which depends parametrically on the solution of the REs and are equivalent to the LNA result. For bimolecular reactions this is not the case since the hierarchy of moment equations obtained from the CME is not closed, i.e., the equations for the means involve the covariances and similarly the equations for the covariances involve higher moments. A systematic approximation of these equations can be achieved using the system size expansion which yields the REs and the LNA in the limit of large volumes [22]. The latter represent a closed system of equations for the first two moments.

### Finite molecule number corrections

Finite molecule number corrections to the LNA can be obtained by considering higher order terms in the sytem size expansion. The latter implies that the moments 〈*ε_k_ε_l_*...*ε_m_*〉 have an expansion of the form [21,55]:

(18) $$\langle \varepsilon_{k}\varepsilon_{l}...\varepsilon_{m}\rangle =\overset{\infty }{\underset{j=0}{\sum }}{\left[\varepsilon_{k}\varepsilon_{l}...\varepsilon_{m}\right]}_{j}\Omega^{-j/2}.$$

For each term in the above expression an closed form equation can be derived [21,22]. In particular to leading order, the mean concentrations and the covariances are given by [*ε_i_*]_0 _= 0 and [*ε_i_ε_j_*]_0 _= *σ_ij _*which is the LNA result. Note that for deterministic initial conditions [*ε_i_ε_j_*]_1 _= 0 as shown in [21] and hence the next to leading order corrections to these quantifies are given by [*ε_i_*]_1 _and [*ε_i_ε_j_*]_2 _which are generally non-zero if bimolecular reactions are considered. In order to relate the above moments back to the moments of the concentration variables we use Eqs. (14) and (18) to find expressions for the mean concentrations and covariance of fluctuations

(19a) $$\langle \frac{n_{i}}{\Omega }\rangle =\left[X_{i}\right]+\Omega^{-1}{\left[\varepsilon_{i}\right]}_{1}+O\left(\Omega^{-2}\right),$$

(19b) $$\begin{array}{llll} \Sigma_{ij} & =\langle \left(\frac{n_{i}}{\Omega }-\langle \frac{n_{i}}{\Omega }\rangle \right)\left(\frac{n_{j}}{\Omega },-,\langle \frac{n_{j}}{\Omega }\rangle \right)\rangle \; & & \;\\ & =\Omega^{-1}{\left[\varepsilon_{i}\varepsilon_{j}\right]}_{0}+\Omega^{-2}\left({\left[\varepsilon_{i}\varepsilon_{j}\right]}_{2}-{\left[\varepsilon_{i}\right]}_{1}{\left[\varepsilon_{j}\right]}_{1}\right)+O\left(\Omega^{-3}\right)\; & & \;\\ & \; & \end{array}$$

Again, the leading order (Ω^0^) contribution to the mean concentrations, Eq. (19a), is given by the macroscopic REs while the leading order contribution given by the Ω^-1 ^term in Eq. (19b) corresponds to the LNA estimate for the variance and the covariance. Including terms to order Ω^-1 ^in Eq. (19a) gives the EMRE estimate of the mean concentrations which corrects the solution of the REs [20]. Finally, considering also the Ω^-2 ^term in Eq. (19b) gives the IOS (Inverse Omega Squared) estimate of the variance and the covariance. From the form of this higher order contribution it is clear that the variance estimate is centered around the EMRE concentrations and is of higher accuracy than the LNA method.

It now follows using definitions (2) and (3) in the main text that the CV and Fano factor have the following expansions in powers of the inverse volume

(20) $$\begin{array}{llll} C_{V,i} & =\frac{\Omega \Sigma_{ii}^{1/2}}{\langle n_{i}\rangle }=C_{V,i}^{\text{LNA}}\left(1+\Omega^{-1}\left(\frac{{\left[\varepsilon_{i}\varepsilon_{i}\right]}_{2}-{\left[\varepsilon_{i}\right]}_{1}^{2}}{\sigma_{ii}}-\frac{1}{2}\frac{{\left[\varepsilon_{i}\right]}_{1}}{\left[X_{i}\right]}\right)+O\left(\Omega^{-2}\right)\right),\; & & \;\\ F_{i} & =\frac{\Omega^{2}\Sigma_{ii}}{\langle n_{i}\rangle }=F_{i}^{\text{LNA}}\left(1+\Omega^{-1}\left(\frac{{\left[\varepsilon_{i}\varepsilon_{i}\right]}_{2}-{\left[\varepsilon_{i}\right]}_{1}^{2}}{\sigma_{ii}}-\frac{{\left[\varepsilon_{i}\right]}_{1}}{\left[X_{i}\right]}\right)+O\left(\Omega^{-2}\right)\right).\; & & \;\\ & \; & \end{array}$$

Again, the leading order contributions are determined by the LNA result, Eq. (17). Note that the factors multiplying Ω^-1 ^in Eq. (20) yield the relative corrections to the LNA measures and are denoted by *c_i _*and *f_i _*in the main text. Note also that each of these factors contains a contribution stemming from a change in the variance of concentration fluctuations and another one reflecting the change in the mean of the concentrations. The equations determining the coefficients [*ε_i_*]_1 _and [*ε_i_ε_i_*]_2 _needed to compute Eqs. (20) have been derived in Refs. [21] and [22]. As shown therein, the quantities depend only on $\left[\vec{X}\right]$ and the vector of reaction rate constants $\vec{k}=\left(k_{1},k_{2},\ldots ,k_{R}\right)$. Hence the CVs and Fano factors depend parametrically on the reaction rate constants through the solution of the REs together with their initial conditions.

In Additional file 5 we have verified the correctness of iNA's implementation of the IOS for the example of a simple enzyme catalyzed reaction against an analytical result obtained in Ref. [22], Eq. (74) therein. We remark that using the IOS it is also possible to deduce the mean concentrations accurately to order Ω ^-2 ^[21] which is superior to the EMRE and hence can be used as an error estimate of the method. However the variance about these concentrations is of Ω^-3 ^as can be seen from Eq. (19b) and hence requires to consider higher orders in the system size expansion.

## Discussion

In this article we have analyzed the parametric dependence of intrinsic noise in gene regulatory networks by means of average concentrations and variances as well as noise measures such as the CV and the Fano factor. The leading order contributions to the average concentrations and variances of the fluctuations as obtained from the system size expansion are given by the deterministic REs and the LNA respectively. The next to leading order contribution are given by novel terms which we have referred to as the EMRE and the IOS approximations respectively. The relative size of these corrections to the LNA are proportional to the inverse compartment volume and to the size of the bimolecular reaction rate constants. Hence, as we have demonstrated, these higher order terms can be significant for networks involving low copy number of molecules and nonlinear reaction kinetics as is common in gene regulation.

In the case of sRNA regulation we have found that for highly expressed proteins the size of the noise can attain a minimum at intermediate stress levels. This result is in line with the LNA's prediction and may perhaps be advantageous as a mechanism of noise minimization in gene expression. The LNA result predicts that such optimization is indeed possible even for low protein expression levels yet the EMRE/IOS analysis and stochastic simulations show that this is not the case, i.e., the CV in the protein fluctuations increases monotonically with the stress levels.

For the case of gene autoregulation we observed that the LNA reliably describes the CV of mRNA and protein fluctuations when the transcription rate, a first order rate constant, is varied but gives very different results from simulations and the EMRE/IOS analysis when the protein-DNA association constant is varied (a bimolecular rate constant measuring the strength of the feedback loop). In particular in the latter case the LNA predicts a maximum in the CV is achieved as one increases the strength of the negative feedback loop whereas the EMRE/IOS analysis shows that the CV increases monotonically. We also found that the LNA can give considerably misleading results for the amplitude and period of noise-induced oscillations in the expression of a single gene while it becomes increasingly more accurate as the gene copy number is increased.

Hence in summary we have shown by means of these examples that the LNA's predictions regarding the regulation of noise by genetic regulatory networks can be quite different than those obtained by stochastic simulations using the SSA. In contrast the results from the EMRE/IOS methods agree well with those obtained from the SSA for the examples studied here. This is surprising since transcriptional feedback involves transitions between internal states of a gene represented by only one or two copies in a cell. We note that the methods presented can become inaccurate when the noise contribution of the feedback loop dominates. However, our methods enjoy the advantage that they can be computed in a fraction of the time needed to calculate the SSA. Hence the EMRE/IOS analysis tools implemented in iNA 0.4 present a quick means to accurately study the stochastic properties of biochemical reaction networks of intermediate or large size involving many bimolecular reactions.

## Availability

**Project name: **intrinsic Noise Analyzer

**Version: **0.4.2

**Project home page: **http://code.google.com/p/intrinsic-noise-analyzer

**Operating systems: **platform independent, binaries available for Mac OSX, Linux and Windows

**Programming language: **C++

**License: **GNU GPL v2

## Competing interests

The authors declare that there are no competing interests.

## Authors' contributions

RG, HM and PT conceived the study, HM and PT implemented the software, PT performed the analysis, HM performed the benchmarks, PT and RG wrote the paper.

## Supplementary Material

Additional file 1 **Coefficients of variation of coding and non-coding transcripts as a function of stress levels**. CV of coding and non-coding transcripts in sRNA regulated gene expression as a function of the stress level ***α***. In (a) we see that the mRNA CV increases while the CV of sRNA decreases as the stress level increases. Notice that the IOS theory "linearizes" the LNA predictions around the crossover point. The predictions are well confirmed by stochastic simulations shown in (b). Click here for file

Additional file 2 **Coefficients of variation of proteins in autoregulated gene expression as a function of feedback strength**. Protein CV of the autoregulated gene expression model is shown as a function of average fraction of repressed promoter states which are a measure of the feedback strength. Unlike the CV of mRNAs, under low copy number conditions the CV of unphosphorylated (a) and phosphorylated proteins (b) predicted by the LNA is in qualitative agreement with the IOS analysis. Notice that the IOS results more closely match those predicted by the SSA. Click here for file

Additional file 3 **Amplification of damped oscillations in a single gene negative feedback loop**. Transient oscillations in the average mRNA and protein levels from negative feedback with a single gene copy number per cell. (a) compares the mean concentrations of the REs and EMREs. The latter predicts an amplification of the damped oscillations which is not captured by the REs. The result is in good agreement with the SSA shown in (b). The parameters used are given in Table 2 except for a volume of 50 × 10^-15^*l*, $\tilde{\Omega }=\text{ 3}0{\left(nM\right)}^{-\text{1}}$ and *k*_0 _= 3 × 10^3^(*nM*)^-1^. Click here for file

Additional file 4 **Improved model editing capabilities in iNA 0.4**. iNA's GUI is equipped with a user friendly model editor. In (a) shows the list of reaction definition along with their propensities. The reaction editor shown in (b) facilitates the creation and editing of reactions through their chemical equation. The propensities are generated automatically using the law of mass action or can be specified explicitly. (c) In addition to the standard SBML format, iNA also supports the convenient SBML shorthand format which allows the specification of the essential model structure using a simple markup language. Click here for file

Additional file 5 **Verification of iNA's implementation**. We have verified the soundness of our implementation by comparison with the analytical result using the IOS derived in Ref. [22]. The graph shows the ratio of the IOS and LNA variance (given by the contributions up to orders Ω^-1 ^and Ω^-2 ^of Eq. (19b), respectively) obtained from the system size expansion (SSE) against the fraction *δ *of free enzyme per total enzyme concentration at steady state. This is also compared to the SSA where the ratio of SSA and LNA variance has been used. Click here for file

## Declarations

The publication costs for this article were funded by the corresponding author.

This article has been published as part of *BMC Genomics *Volume 14 Supplement S4, 2013: Selected articles from the IEEE International Conference on Bioinformatics and Biomedicine 2012: Genomics. The full contents of the supplement are available online at http://www.biomedcentral.com/bmcgenomics/supplements/14/S4.
