# Supplementary figures and images for: How reliable is the linear noise approximation of gene regulatory networks?
Source: BMC Genomics. 2013 Oct 1;14(Suppl 4):S5. doi: 10.1186/1471-2164-14-S4-S5 (PMC3849541; doi:10.1186/1471-2164-14-S4-S5)

Coefficient of variation (LNA &amp; IOS)

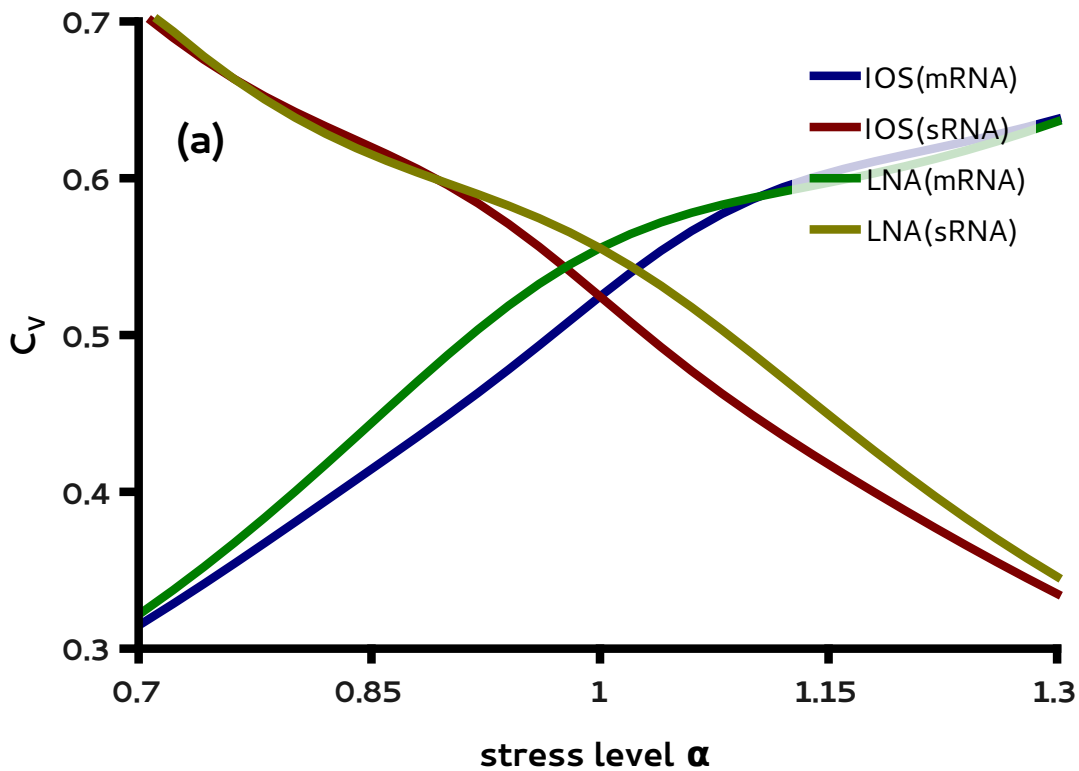

Coefficient of variation (SSA)

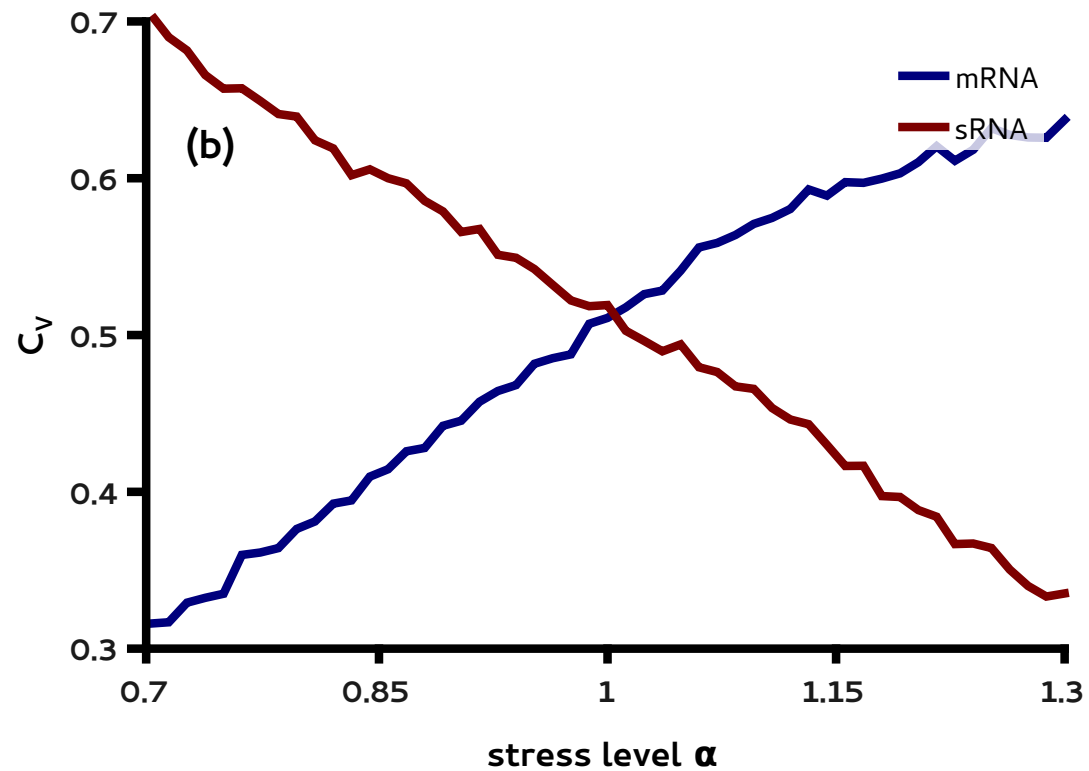

Supplement: Additional file 1 — Coefficients of variation of coding and non-coding transcripts as a function of stress levels. CV of coding and non-coding transcripts in sRNA regulated gene expression as a function of the stress level α. In (a) we see that the mRNA CV increases while the CV of sRNA decreases as the stress level increases. Notice that the IOS theory "linearizes" the LNA predictions around the crossover point. The predictions are well confirmed by stochastic simulations shown in (b). [file 1471-2164-14-S4-S5-S1.pdf]

Coefficient of variation

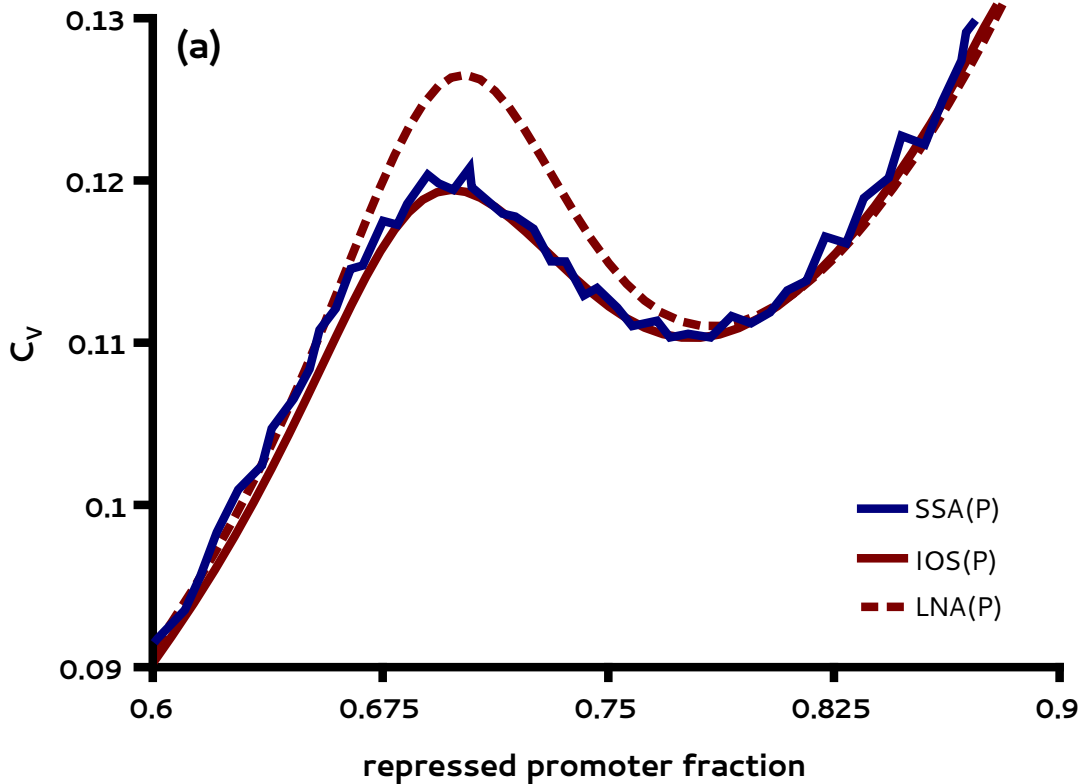

Coefficient of variation

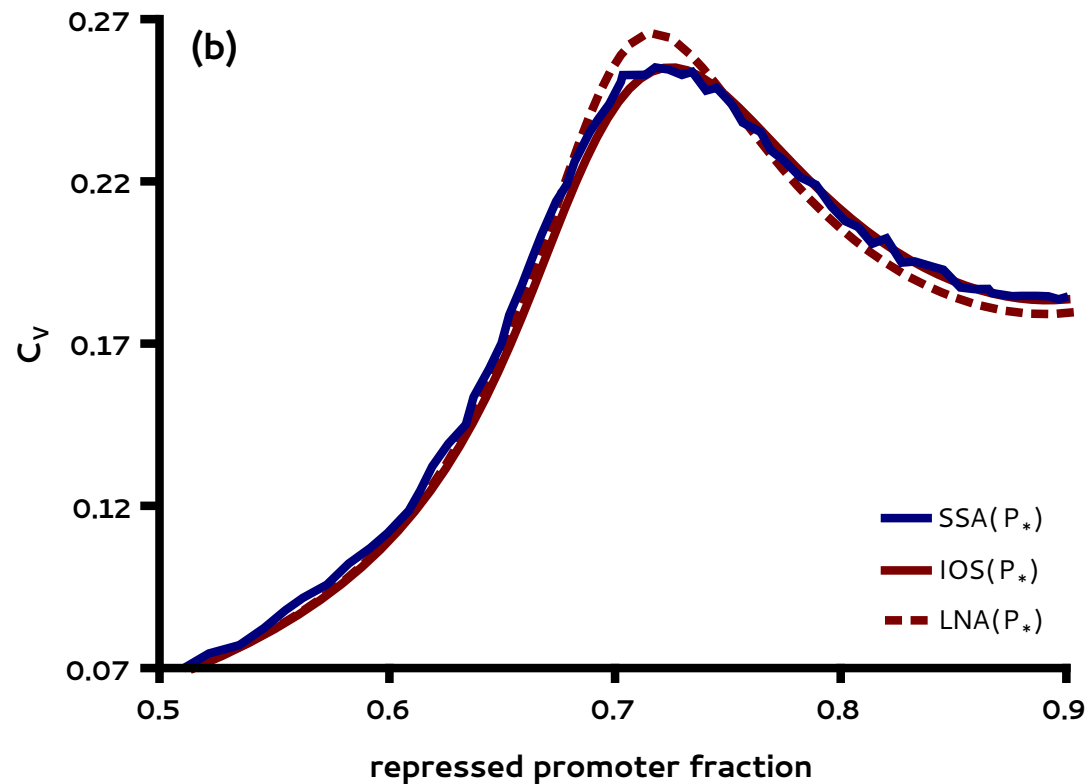

Supplement: Additional file 2 — Coefficients of variation of proteins in autoregulated gene expression as a function of feedback strength. Protein CV of the autoregulated gene expression model is shown as a function of average fraction of repressed promoter states which are a measure of the feedback strength. Unlike the CV of mRNAs, under low copy number conditions the CV of unphosphorylated (a) and phosphorylated proteins (b) predicted by the LNA is in qualitative agreement with the IOS analysis. Notice that the IOS results more closely match those predicted by the SSA. [file 1471-2164-14-S4-S5-S2.pdf]

# Mean concentrations (EMRE & IOS)

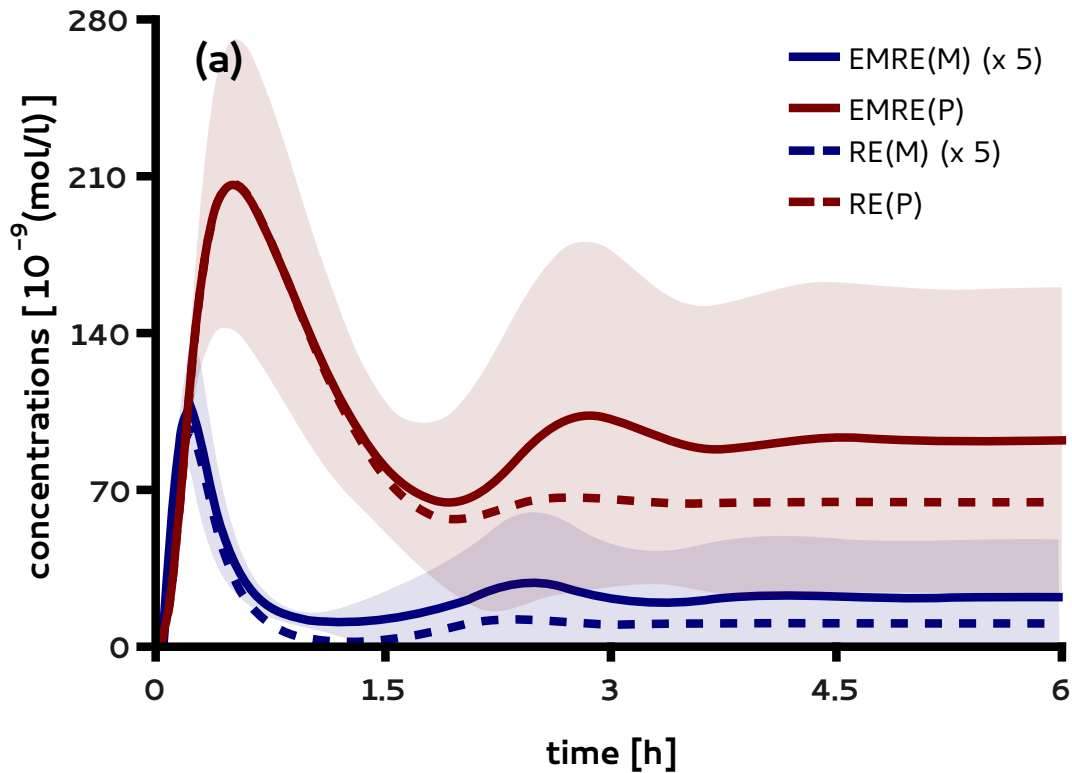

# Stochastic Simulation Algorithm

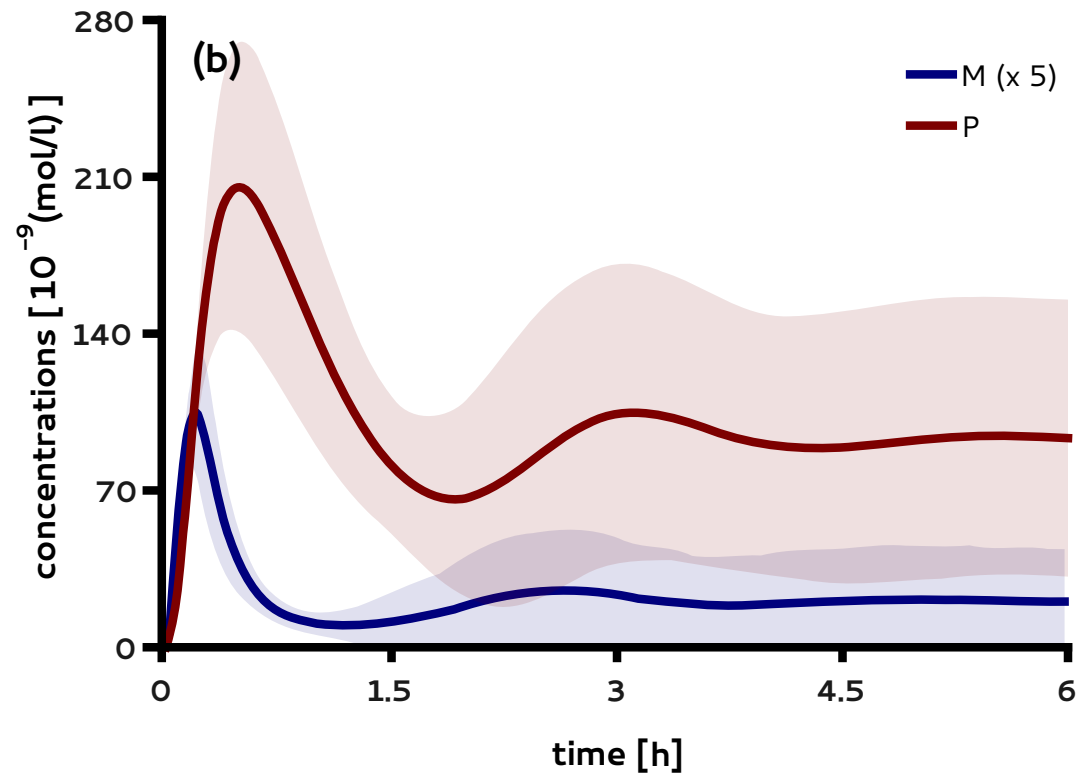

Supplement: Additional file 3 — Amplification of damped oscillations in a single gene negative feedback loop. Transient oscillations in the average mRNA and protein levels from negative feedback with a single gene copy number per cell. (a) compares the mean concentrations of the REs and EMREs. The latter predicts an amplification of the damped oscillations which is not captured by the REs. The result is in good agreement with the SSA shown in (b). The parameters used are given in Table 2 except for a volume of 50 × 10-15l, Ω˜= 30nM-1 and k0 = 3 × 10^3^(nM)-1. [file 1471-2164-14-S4-S5-S3.pdf]

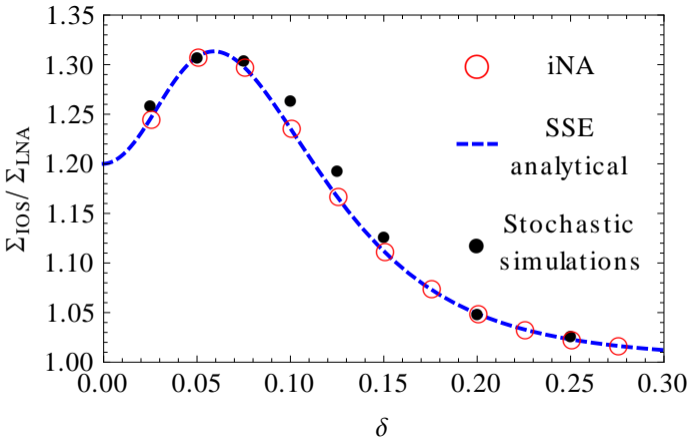

Supplement: Additional file 5 — Verification of iNA's implementation. We have verified the soundness of our implementation by comparison with the analytical result using the IOS derived in Ref. [22]. The graph shows the ratio of the IOS and LNA variance (given by the contributions up to orders Ω-1 and Ω-2 of Eq. (19b), respectively) obtained from the system size expansion (SSE) against the fraction δ of free enzyme per total enzyme concentration at steady state. This is also compared to the SSA where the ratio of SSA and LNA variance has been used. [file 1471-2164-14-S4-S5-S5.pdf]
