# Supplementary material for: How reliable is the linear noise approximation of gene regulatory networks?
Source: BMC Genomics. 2013 Oct 1;14(Suppl 4):S5. doi: 10.1186/1471-2164-14-S4-S5 (PMC3849541; doi:10.1186/1471-2164-14-S4-S5)
Supplement: Additional file 4 — Improved model editing capabilities in iNA 0.4. iNA's GUI is equipped with a user friendly model editor. In (a) shows the list of reaction definition along with their propensities. The reaction editor shown in (b) facilitates the creation and editing of reactions through their chemical equation. The propensities are generated automatically using the law of mass action or can be specified explicitly. (c) In addition to the standard SBML format, iNA also supports the convenient SBML shorthand format which allows the specification of the essential model structure using a simple markup language. [file 1471-2164-14-S4-S5-S4.pdf]

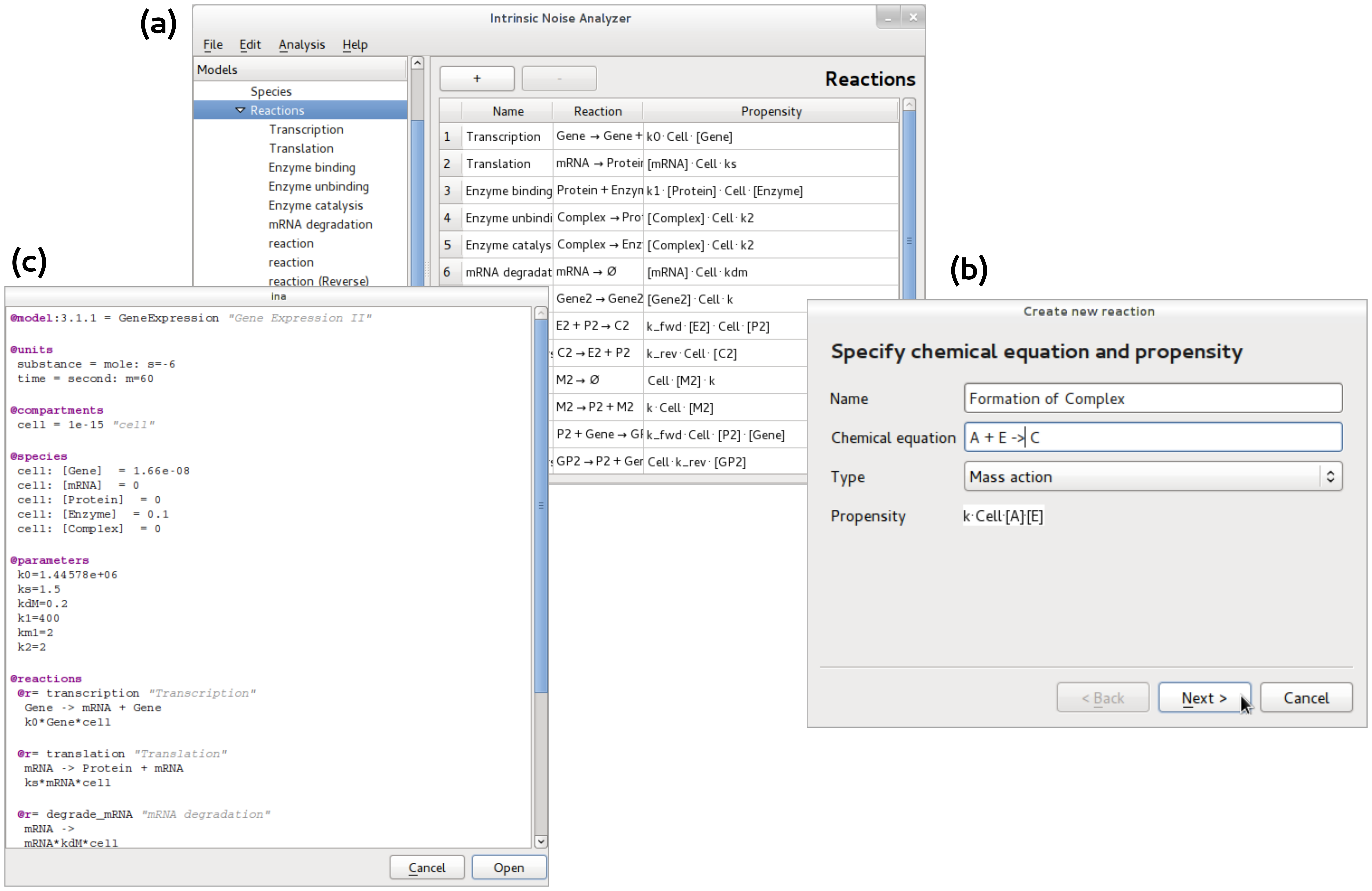
(a)
Intrinsic Noise Analyzer
File Edit Analysis Help
Models
Species
Reactions
Transcription
Translation
Enzyme binding
Enzyme unbinding
Enzyme catalysis
mRNA degradation
reaction
reaction
reaction (Reverse)
+
-
Reactions
Name Reaction Propensity
1 Transcription Gene → Gene + k0 · Cell · [Gene]
2 Translation mRNA → Protei [mRNA] · Cell · ks
3 Enzyme binding Protein + Enzyn k1 · [Protein] · Cell · [Enzyme]
4 Enzyme unbindi Complex → Pro [Complex] · Cell · k2
5 Enzyme catalys Complex → Enz [Complex] · Cell · k2
6 mRNA degradat mRNA → Ø [mRNA] · Cell · kdm
Gene2 → Gene2 [Gene2] · Cell · k
E2 + P2 → C2 k_fwd · [E2] · Cell · [P2]
C2 → E2 + P2 k_rev · Cell · [C2]
M2 → Ø Cell · [M2] · k
M2 → P2 + M2 k · Cell · [M2]
P2 + Gene → GI k_fwd · Cell · [P2] · [Gene]
GP2 → P2 + Ger Cell · k_rev · [GP2]
(b)
Create new reaction
Specify chemical equation and propensity
Name
Formation of Complex
Chemical equation
A + E -> C
Type
Mass action
Propensity
k·Cell·[A]·[E]
< Back
Next >
Cancel
(c)
ina
@model:3.1.1 = GeneExpression "Gene Expression II"
@units
substance = mole: s=-6
time = second: m=60
@compartments
cell = 1e-15 "cell"
@species
cell: [Gene] = 1.66e-08
cell: [mRNA] = 0
cell: [Protein] = 0
cell: [Enzyme] = 0.1
cell: [Complex] = 0
@parameters
k0=1.44578e+06
ks=1.5
kdM=0.2
k1=400
km1=2
k2=2
@reactions
@r= transcription "Transcription"
Gene -> mRNA + Gene
k0*Gene*cell
@r= translation "Translation"
mRNA -> Protein + mRNA
ks*mRNA*cell
@r= degrade_mRNA "mRNA degradation"
mRNA ->
mRNA*kdM*cell
Cancel
Open
